# More Time Spent in Sedentary Behaviors is Associated with Higher Plantar Pressures in Older Women

**DOI:** 10.3390/ijerph17062089

**Published:** 2020-03-21

**Authors:** Mario Kasović, Lovro Štefan, Martin Zvonař

**Affiliations:** 1 Faculty of Kinesiology, Department of General and Applied Kinesiology, University of Zagreb, 10 000 Zagreb, Croatia; mario.kasovic@kif.hr; 2 Faculty of Sports Studies, Masaryk University, 625 00 Brno, Czech Republic; zvonar@fsps.muni.cz; 3 RECETOX, Faculty of Science, Masaryk University, 625 00 Brno, Czech Republic

**Keywords:** sedentariness, foot, forces, relation

## Abstract

*Background:* Although obesity has been consistently correlated with higher plantar pressure during the lifespan, to date little evidence has been provided regarding of how domain-specific and total sedentary behaviors may be correlated with plantar pressures. Moreover, high peak plantar pressures have been consistently associated with foot pain and discomfort, which prevent individuals from being physically active. Therefore, the main purpose of the study was to explore the correlations between time spent in sedentary behaviors and plantar pressures. *Methods:* We recruited 120 older women aged ≥60 years. To assess the time spent in different domains of sedentary behavior, we used the Measure of Older Adults’ Sedentary Time (MOST) questionnaire. Peak pressures beneath forefoot, midfoot and hindfoot were measured with a Zebris pressure platform. *Results:* In the unadjusted model, peak pressures were significantly correlated with almost all domain-specific sedentary behaviors (*r* = 0.15–0.41). Total time spent in sedentary behaviors was significantly correlated with forefoot (*r* = 0.40, *p* < 0.001), hindfoot (*r* = 0.31, *p* < 0.001) and total peak plantar pressure (*r* = 0.40, *p* < 0.001). In a model adjusted for age, the risk of falls, foot pain and gait velocity, similar significant correlations between sedentary behaviors and plantar pressures remained. *Conclusions*: Our study shows moderate correlation between domain-specific and total time spent in sedentary behaviors and plantar pressure beneath different foot regions in a sample of older women.

## 1. Introduction

Time spent in sedentary behaviors has become one of the primarily issues in today’s society. Sedentary behaviors are defined as “any waking behavior characterized by energy expenditure ≤1.5 metabolic equivalents, while in a sitting, reclining or lying posture” [1]. Independent of physical activity, sedentary behaviors have often been associated with negative health outcomes, including overweight/obesity status, elevated blood pressure and total cholesterol, lower levels of self-esteem, physical fitness and academic achievement [2].

In the last 50 years, the population of people aged ≥60 years has increased by 2%, with estimates that the number will increase to 22% by 2050 [3]. Older adults are facing many health-related consequences, including twice as many disabilities and four times as many physical limitations as people who are aged <60 years [4]. Despite well-documented health benefits of physical activity, 60%–70% of older adults spend their waking hours in sedentary behaviors [5].

One potential limitation of participating in regular physical activity and spending more time being sedentary comes from deviated foot structure and function that older adults suffer from. Biomechanically, the foot needs to perform diverse functions, especially during weight-bearing activities [6]. Such activities may lead to pain and discomfort and potentially discourage individuals from being physically active [7]. Foot pain has often been correlated with higher plantar pressures [7]. Studies conducted among children have shown that being sedentary is correlated with higher plantar pressures under 2–5 toes [6]. To date, we found no study examining the correlations between different domains of sedentary behavior and plantar pressures in older adults. According to previous studies, only 25% of older adults meet the recommended levels of physical activity and the majority of them spend most of the time being sedentary [2]. Since older adults have deviated structure and function of the foot [7] and are physically inactive [5], it is necessary to explore whether such behaviors may be correlated with higher plantar pressures beneath different foot regions. Additionally, such associations may be able to help future strategies and policies for creating a set of activities designed for older individuals with higher plantar pressures and significant foot pain.

Therefore, the main purpose of the study was to explore the correlations between time spent in sedentary behaviors and plantar pressures.

## 2. Materials and Methods

### 2.1. Study Participants

One-hundred and twenty older women aged ≥60 years participated in this cross-sectional study. More detailed information about the methodology and sample size collection has been reported previously [8].

All procedures performed in this study were anonymous and according to Declaration of Helsinki, also approved by the Faculty of Kinesiology, University of Zagreb, Croatia (Ethical code number: 2019). Additionally, all participants had given a written informed consent for participation in the study.

### 2.2. Dynamic Plantar Pressure

To assess the level of peak plantar pressure, we used a Zebris plantar pressure platform (FDM; GmbH, Munich, Germany; number of sensors: 11,264; sampling rate: 100 Hz; sensor area: 149 cm × 54.2 cm). The dynamic mode methodology is described in detail elsewhere [8]. The software generated the data regarding peak plantar pressures beneath the different foot regions of forefoot, midfoot and hindfoot.

### 2.3. Sedentary Behaviors

To assess sedentary behavior, we used the Measure of Older Adults’ Sedentary Time questionnaire [9]. More specific details regarding the questionnaire in terms of scoring protocol and psychometric properties (reliability and validity) have been reported elsewhere [9]. For the purpose of this study, we created domain-specific categories of sedentary behavior as follows: (1) screen-time (watching television and using a computer/tablet), (2) reading, (3) socializing, (4) transportation and (5) hobbies and other. Total sedentary behavior time was calculated by summing all domain-specific categories. The internal consistency of the questionnaire in our study was good (Cronbach’s alpha = 0.82).

### 2.4. Covariates

Chronological age was calculated by using participants’ years of birth and the present year. To assess risk of falls, we used the Downtown Fall Risk Index [10], a reliable and valid instrument to measure five modules as follows: (1) previous falls, (2) medication, (3) sensory deficits, (4) mental state and (5) gait. This results in 11 different risk factors, which are then summarized and given a score between 0 and 11. A higher score indicates a higher risk of falls [10]. Of note, the internal consistency of the questionnaire in our study was satisfactory (Cronbach’s alpha = 0.72). Presence of foot pain was determined according to a previously used question: “On most days do you have pain, aching, or stiffness in either of your feet?” [11]. Responses were (1) “No”; (2) “Yes, left foot only”; (3) “Yes, right foot only”; (4) “Yes, both feet”; (5) “Yes, not sure what side” and (6) “Unknown”. For this analysis, the responses “Yes, left foot only”, “Yes, right foot only”, “Yes, both feet" and “Yes, not sure what side” were collapsed into a “Yes” vs. “No” category. Of note, none of the participants responded with the “Unknown” response. In addition, gait velocity was objectively assessed using Zebris software, which generated the data in km/h.

### 2.5. Data Analysis

Basic descriptive statistics are presented as mean ± SD or median (25th–75th percentile range) for normally and not normally distributed variables in Table 1. For further analyses, we chose the plantar pressures for right foot, since *t*–tests for dependent samples showed no significant differences between the feet. To assess the normality, we used the Kolmogorov–Smirnov normality test. Since the time spent in sedentary behavior was not normally distributed, the correlations between all sedentary behavior domains and total sedentary behavior and plantar pressures beneath different foot regions were calculated by using Spearman’s rank of correlation (*r*). In an unadjusted model, we only calculated the aforementioned correlations (Table 2). We additionally adjusted for age, the risk of falls, foot pain and gait velocity (Table 3). We used the Statistical Packages for Social Sciences (SPSS Inc., Chicago, IL, USA) program with a statistical significance of *p* < 0.05 to calculate the relations.

## 3. Results

Basic descriptive statistics of the study participants are presented in Table 1. The mean age was slightly above the age of 70 years, while the highest peak plantar pressures were detected beneath the forefoot region, followed by the hindfoot and midfoot regions. Older individuals spent most of the time in front of the screen, socializing and reading, with almost 7 h spent in total sedentary behavior.

The correlations between domain-specific sedentary behaviors and total sedentary behavior with different plantar pressures are presented in Table 2. The coefficients ranged between 0.01 and 0.44, where screen-time was the strongest predictor of forefoot, midfoot and hindfoot plantar pressures, followed by total sedentary behavior and reading. When the model was adjusted for age, the risk of falls, foot pain and gait velocity (Table 3), similar significant correlations between domain-specific sedentary behaviors and total sedentary behavior with different plantar pressures were observed.

## 4. Discussion

The main purpose of the study was to explore the correlations between time spent in sedentary behaviors and plantar pressure. Our main findings are as follows: (1) More time spent in different sedentary behavior domains and total sedentary behavior is correlated with higher peak pressures in forefoot, midfoot and hindfoot regions of the foot and total peak plantar pressure; and (2) when we adjusted for age, the risk of falls, foot pain ad gait velocity, more time spent in different sedentary behavior domains and total sedentary behavior remained significantly correlated with higher peak pressures in forefoot, midfoot and hindfoot regions of the foot and total peak plantar pressure.

This is the first study exploring the correlations between time spent in sedentary behaviors and plantar pressure in older adults. Studies have shown that high peak plantar pressures have been consistently associated with foot pain and discomfort, which prevents individuals from being physically active [10,11,12]. In light of our findings, one previous study conducted among children has shown that the percentage of sedentary behavior is significantly correlated with peak plantar pressure in the toe region only in girls (*r* = 0.53, *p* = 0.04) [6]. In general, accumulating evidence has shown that older adults have somewhat different foot structure and function, including flatter foot, intrinsic foot muscle weakness, altered plantar pressure loading patterns during walking and reduced plantar tactile sensitivity [12]. Since more time spent in sedentary behaviors leads to higher body-mass index [13] and obesity is correlated with altered plantar pressure distribution in older adults [14], our findings are somewhat expected. It should be noted that we did not collect additional data regarding foot structure, including flatness, soft tissue thickness and arch characteristics, which have been previously correlated with obesity and plantar pressure [14]. In line with that, during overloading conditions, the vertical forces and pressures generated beneath different regions of the foot contribute to the collapse of the medial longitudinal arch, causing a greater contact area of the foot with the ground [15]. Additionally, older individuals who spent more time in sedentary behaviors tend to have lower muscle mass [16], which also contributes to foot flattening [14]. In terms of foot function, previous studies have shown that obese individuals walk more slowly, with shorter and wider steps and longer stance phase duration [17], which may cause certain pain and discomfort and, in addition, higher levels of plantar pressure.

The significance of our study lies in the fact that we detected moderate correlations between the time spent in different domains and total sedentary behavior and peak plantar pressures beneath different foot regions. Although we cannot determine the causality of the correlation, future studies should use objective measures to assess the time spent in sedentary behavior. Additionally, more detailed health-related characteristics of the participants and the presence of chronic conditions need to be collected. In that way, we will be able to discriminate the correlations between healthy and less healthy individuals.

This study has several limitations. We used a cross-sectional design, which enabled us to determine the causality of the correlations. Additionally, our findings are based and determined on a relatively small sample of participants, who were healthy with no disease reports at the time the study was conducted. Therefore, it is possible that the correlations would have been different when including less healthy older adults. Next, we based our study on a sample living in the urban part of the country, speaking Croatian and only the White race. Finally, by including men, we could be able to observe sex-specific correlations between the sedentary behavior and peak plantar pressure.

## 5. Conclusions

In conclusion, our study shows that more time spent in domain-specific and total sedentary behaviors is correlated with higher peak plantar pressures under the forefoot, midfoot and hindfoot plantar areas and with average peak plantar pressure, even after adjusting for several potential covariates. This is particularly important, because higher levels of peak plantar pressures are often associated with pain and discomfort in lower extremities, which may lead to being sedentary. Thus, policies and strategies that reduce the time spent in sedentary behaviors and increase the time in organized physical activity should be implemented within the community where they live. Additionally, by understanding the function and structure of the foot in older adults, we could create specific-based activities, which do not lead to pain and discomfort and, in addition, to higher levels of plantar pressure. Such non-weight-bearing activities, like swimming, riding a stationary bike or doing stretching should be incorporated into their leisure-time to decrease the time spent in sedentary behaviors.

## Figures and Tables

**Table 1:** Basic descriptive statistics of the study participants (*n* = 120).

Study Variables	Mean ± SD
Age (years)	71.01 ± 6.77
Height (cm)	158.92 ± 21.41
Weight (kg)	70.29 ± 12.97
Plantar pressure beneath forefoot (N/cm^2^)	46.73 ± 10.65
Plantar pressure beneath midfoot (N/cm^2^)	16.94 ± 7.41
Plantar pressure beneath hindfoot (N/cm^2^)	31.57 ± 7.44
Average peak plantar pressure (N/cm^2^)	31.75 ± 6.15
Screen-time (h/d) *	2.18 (0.70–4.00)
Reading (h/d) *	1.00 (0.36–2.00)
Socializing (h/d) *	1.33 (0.57–2.14)
Transportation (h/d) *	0.57 (0.14–1.14)
Hobbies (h/d) *	0.92 (0.43–2.42)
Total sedentary behavior (h/d) *	6.83 (3.57–10.31)
Downtown Fall Risk Index *	2.00 (1.00–3.00)
Foot pain (Yes/No, %) **	53/47
Gait velocity (km/h)	3.00 ± 1.00

* denotes using median (25th–75th percentile range); ** denotes using percentage value.

**Table 2:** Unadjusted correlations between sedentary behavior domains and total sedentary behavior with peak plantar pressures (*n* = 120).

Study Variables	Forefoot	Midfoot	Hindfoot	Total Pressure
	*r* (*p*-Value)	*r* (*p*-Value)	*r* (*p*-Value)	*r* (*p*-Value)
Screen-time	0.41 (<0.001)	0.23 (0.014)	0.31 (<0.001)	0.44 (<0.001)
Reading	0.32 (<0.001)	0.15 (0.100)	0.30 (<0.001)	0.35 (<0.001)
Socializing	0.26 (0.004)	0.08 (0.387)	0.25 (0.005)	0.29 (<0.001)
Transportation	0.26 (0.004)	0.14 (0.138)	0.25 (0.005)	0.28 (0.002)
Hobbies	0.24 (0.007)	0.01 (0.905)	0.16 (0.081)	0.20 (0.028)
Total sedentary behavior	0.40 (<0.001)	0.13 (0.157)	0.31 (<0.001)	0.40 (<0.001)

*p* < 0.05.

**Table 3:** Adjusted * correlations between sedentary behavior domains and total sedentary behavior with peak plantar pressures (*n* = 120).

Study Variables	Forefoot	Midfoot	Hindfoot	Total Pressure
	*r* (*p*-Value)	*r* (*p*-Value)	*r* (*p*-Value)	*r* (*p*-Value)
Screen-time	0.41 (<0.001)	0.24 (0.008)	0.32 (<0.001)	0.48 (<0.001)
Reading	0.29 (<0.001)	0.23 (0.011)	0.33 (<0.001)	0.41 (<0.001)
Socializing	0.23 (0.012)	0.04 (0.639)	0.23 (0.014)	0.25 (0.006)
Transportation	0.34 (<0.001)	0.27 (0.004)	0.28 (0.002)	0.43 (<0.001)
Hobbies	0.20 (0.034)	0.01 (0.970)	0.14 (0.135)	0.18 (0.050)
Total sedentary behavior	0.40 (<0.001)	0.20 (0.035)	0.34 (<0.001)	0.46 (<0.001)

* correlations adjusted for age, the risk of falls, foot pain and gait velocity; *p* < 0.05.
